# An open spatial capture–recapture model for estimating density, movement, and population dynamics from line‐transect surveys

**DOI:** 10.1002/ece3.7566

**Published:** 2021-05-03

**Authors:** Timothy A. Gowan, Nathan J. Crum, Jason J. Roberts

**Affiliations:** ^1^ Fish and Wildlife Research Institute Florida Fish and Wildlife Conservation Commission St. Petersburg FL USA; ^2^ Department of Wildlife Ecology and Conservation University of Florida Gainesville FL USA; ^3^ Marine Geospatial Ecology Laboratory Duke University Durham NC USA

**Keywords:** density estimation, distance sampling, hierarchical model, population dynamics, right whale, spatial capture–recapture

## Abstract

The purpose of many wildlife population studies is to estimate density, movement, or demographic parameters. Linking these parameters to covariates, such as habitat features, provides additional ecological insight and can be used to make predictions for management purposes. Line‐transect surveys, combined with distance sampling methods, are often used to estimate density at discrete points in time, whereas capture–recapture methods are used to estimate movement and other demographic parameters. Recently, open population spatial capture–recapture models have been developed, which simultaneously estimate density and demographic parameters, but have been made available only for data collected from a fixed array of detectors and have not incorporated the effects of habitat covariates. We developed a spatial capture–recapture model that can be applied to line‐transect survey data by modeling detection probability in a manner analogous to distance sampling. We extend this model to a) estimate demographic parameters using an open population framework and b) model variation in density and space use as a function of habitat covariates. The model is illustrated using simulated data and aerial line‐transect survey data for North Atlantic right whales in the southeastern United States, which also demonstrates the ability to integrate data from multiple survey platforms and accommodate differences between strata or demographic groups. When individuals detected from line‐transect surveys can be uniquely identified, our model can be used to simultaneously make inference on factors that influence spatial and temporal variation in density, movement, and population dynamics.

## Graphical abstract

We developed an open spatial capture–recapture model that can be applied to line‐transect survey data. The model estimates demographic parameters while simultaneously providing inference on factors that influence spatial and temporal variation in density and space use. We illustrate its use with aerial survey data for North Atlantic right whales (*Eubalaena glacialis*) in the southeastern United States.

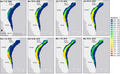

## 1 INTRODUCTION

Estimating population size is of primary interest in many ecological studies and is fundamental to assessing the status of wildlife populations. Estimates of local abundance or density (i.e., abundance per unit area) are also required to characterize the spatial distribution of a population and inform management decisions, such as configuring protection zones for endangered species and prioritizing sites for the eradication of invasive species (Taylor & Hastings, 2004; Udell et al., 2019). Likewise, an understanding of population dynamics and processes that influence temporal variation in abundance can be used to test ecological theory, inform the timing of management actions, and predict the outcome of alternative management strategies (Williams et al., 2002).

Line‐transect sampling is commonly used to survey populations and, when combined with distance sampling methods, can provide estimates of abundance when detection is imperfect. In distance sampling, the distances to detected animals from a transect line are measured, and detection probability is modeled as a decreasing function of distance (Buckland et al., 2001). Distance sampling can also be applied in modeling spatial variation in density as a function of habitat covariates (Miller et al., 2013). However, the conventional distance sampling assumption that detection probability is 1 for animals located directly on the transect may not hold true for cryptic or aquatic species. Additionally, while repeated surveys can provide estimates of temporal trends in abundance (Moore & Barlow, 2011), distance sampling does not typically provide direct inference on underlying demographic parameters (e.g., recruitment, survival, movement) that drive changes in abundance over time and are often the target of management actions. Open population distance sampling models have been developed to assess population dynamics, but in practice they are generally limited to estimating population growth rate and not the underlying processes of recruitment and survival (Schmidt & Rattenbury, 2018; Sollmann et al., 2015). Similarly, in dynamic *N*‐mixture models, which rely on replicated surveys to estimate abundance and demographic parameters of unmarked populations (Dail & Madsen, 2011), survival and recruitment are often confounded, resulting in less accurate estimates compared with capture–recapture studies (Zipkin et al., 2014).

Capture–recapture methods, in contrast, rely on the recapture of identifiable individuals over time and can provide estimates of abundance as well as demographic parameters for open populations (Williams et al., 2002). Spatial capture–recapture models extend these methods by incorporating the capture locations of individuals, which allows for inference on spatial variation in density and the ability to account for heterogeneity in detection probability due to the proximity of individuals to surveyed locations (Borchers & Marques, 2017; Royle et al., 2018). Most spatial capture–recapture models, however, have been developed for data collected from a fixed array of detectors (e.g., camera traps, hair snares), in which detections are restricted to discrete points in space (Gardner et al., 2010; Royle et al., 2013).

Royle et al. (2011) presented a search‐encounter spatial capture–recapture model to accommodate data collected along a survey path, including a set of line transects. This model allows detections to be anywhere in continuous space and, unlike other spatial capture–recapture and distance sampling models, includes an explicit model for individual movement. Borchers et al. (2015) illustrated that incorporating additional information about an individual's true location can improve inference in spatial capture–recapture models relative to when capture locations are restricted to the location of detectors. In their formulation, Royle et al. (2011) discretized survey transects as a set of regularly spaced points and modeled detection probability as the cumulative hazard to detection over all points. However, the spacing of points chosen by the analyst results in a trade‐off between long computation times (as when the survey path is represented as many points spaced closely together) and biased estimates of the effect of distance on detection probability (as when points are spaced far apart and detection distances are overestimated).

Here, we adapt the search‐encounter spatial capture–recapture model using an approach that is analogous to distance sampling and can be applied to line‐transect survey data. Specifically, we model detection probability as a function of the distance between an individual's location and the closest point on the closest survey transect, which should be more computationally efficient than the cumulative hazard approach (Kéry & Royle, 2020). This model is further extended to estimate demographic parameters (e.g., recruitment and survival, or immigration and emigration) using an open population framework, model variation in density across space and time, model movement as a function of habitat covariates, and accommodate differences between strata or groups.

We illustrate this model using photo‐identification data of North Atlantic right whales *Eubalaena glacialis* collected from aerial line‐transect surveys in a migratory habitat to estimate density, arrival timing, and departure timing. As for many other endangered species, primary conservation measures for right whales include time‐area restrictions on human activities, such as fishing and shipping (Crum et al., 2019; Farmer et al., 2016). Assessing the conservation value of such protection zones and quantifying the cumulative impact of external stressors requires spatially and temporally explicit estimates of absolute abundance, as well as estimates of residence time in an area (Pirotta et al., 2018). Using the right whale case study, we demonstrate how our model can provide these estimates while also providing insight on movement, resource selection, and the effectiveness of monitoring programs. We discuss the applicability to other taxa and further extensions of the model framework.

## 2 METHODS

### 2.1 Study design

The model can be applied to studies where transect lines are surveyed during T separate primary sampling periods. The population is considered open (to recruitment, deaths, and emigration) between each primary period. A robust sampling design (wherein multiple surveys are conducted within each primary period, during which the population is assumed closed; Pollock, 1982) is not required but is expected to improve precision of model estimates and allows inference on movement during primary periods. The number and configuration of transects may vary across sampling occasions, but the region of inference (i.e., state–space) is constant. The locations of transect lines (e.g., start and end points) surveyed on each sampling occasion are recorded. When individuals in the population are detected along a transect, their location is recorded and they are uniquely identified. An individual's location may be recorded with a GPS device or estimated from the sighting distance (e.g., using a rangefinder or inclinometer) and sighting angle. Methods for identifying individuals are varied but include artificial tags and branding (Smout et al., 2011), photo‐identification via natural marks (Hammond, 2009), and genotyping using biopsy or noninvasive sampling (Constantine et al., 2012). Like other standard capture–recapture models, it is assumed that identifying marks are not lost or overlooked.

The model estimates the effects of habitat covariates on density and movement. Covariate values may vary between primary sampling periods, but they must be available or interpolated for the entire spatial extent of the state–space. A modified model without habitat covariates is easily implemented in our framework, but the increased availability of remote sensing and other geospatial data warrants their consideration.

### 2.2 Open population model

To model population dynamics, we used an open population capture–recapture model, following a superpopulation formulation of the Jolly–Seber model (Schwarz & Arnason, 1996). This model estimates probabilities of recruitment to a population, γ, and of survival, ϕ, but in the right whale example, we interpreted γ as the probability of arriving in the study area and ϕ as the probability of persisting in the study area (Krzystan et al., 2018; Lyons et al., 2016). A Bayesian state–space formulation of this model was used to estimate the state variable $z_{i,t}$, where $z_{i,t}=1$ if individual i was in the population (or study area) during primary sampling period t∈T and $z_{i,t}=0$ if it was not (Kéry & Schaub, 2012; Royle & Dorazio, 2009). To implement this model, we used data augmentation, which augments the number of observed individuals, n, with M‐n hypothetical individuals and is equivalent to a discrete $\text{Uniform}\left(0,M\right)$ prior for abundance at t=1 (Royle & Dorazio, 2009).

Here, $\gamma_{1}$ is the proportion of M individuals that are in the population at t=1. For t>1,

$$z_{i,t}∼\text{Bernoulli}\left(\phi_{t‐1}z_{i,t‐1}+\gamma_{t}\left(1‐z_{i,t‐1}\right)\right).$$

Thus, an individual in the population at t‐1 remains in the population through t with probability $\phi_{t‐1}$, and an individual not in the population at t‐1 may enter the population with probability $\gamma_{t}$. Note that, here, $\gamma_{t}$ is conditioned on the number of M individuals that are available to be recruited (i.e., where $z_{i,t‐1}=0$; Gardner et al., 2010). Unlike other formulations of the Jolly–Seber model in which an individual can be recruited only once, here an individual may enter (and leave) the population multiple times during the study, and the probabilities of re‐entry are the same as those for initial entry; this can be modified to restrict individuals to a single entry (by tracking $z_{i,1:t‐1}$; Kéry & Schaub, 2012) or to allow probabilities of re‐entry to differ from initial entry (by tracking $z_{i,1:t‐1}$ and including additional parameters). With this formulation, abundance (the number of individuals in the population) at time t, $N_{t}$, is derived as

$$N_{t}=\sum_{i=1}^{M}z_{i,t}$$

and the number of recruits (individuals entering the population), $R_{t}$, is derived as

$$R_{t}=\sum_{i=1}^{M}\left(1‐z_{i,t‐1}\right)z_{i,t}.$$

We constructed this model to accommodate a robust sampling design (Pollock, 1982), wherein data are collected for $K_{t}$ secondary sampling occasions within a primary sampling period t. The population is assumed closed to recruitment, mortality, and departure across secondary sampling occasions within a primary period but open across primary periods. For the right whale application, we used data collected in the southeastern United States during the 2009–2010 winter, and we defined T=8 primary periods (1–15 December 2009; 16–31 December 2009; 1–15 January 2010; …; 16–31 March 2010), with $K_{t}$ reflecting the number of days on which surveys were flown in each primary period. Following Krzystan et al. (2018), we modeled arrival probability as a quadratic function of time,

$$\text{logit}\left(\gamma_{t}\right)=\beta_{\gamma 1}+\beta_{\gamma 2}time_{t}+\beta_{\gamma 3}time_{t}^{2},$$

and we modeled persistence probability as a linear function of time,

$$\text{logit}\left(\phi_{t}\right)=\beta_{\phi 1}+\beta_{\phi 2}time_{t}.$$

The logit function was used to constrain probabilities between 0 and 1, and time is the vector of primary period indices   t=1, 2, ..., T standardized to mean = 0 and standard deviation = 1.

### 2.3 Density model

In open spatial capture–recapture models, each individual in the population has a latent activity or home range center, $s_{i,t}$, that is defined by its *x*‐ and *y*‐coordinates in space and may vary across primary periods. Due to the high mobility of right whales, we assumed that their activity centers were independent across primary periods, but activity centers could alternatively be modeled to follow a random walk or to be constant across primary periods (Gardner et al., 2018). For a given state–space region, S, the expected density of activity centers, $D_{t}=N_{t}/area\left(S\right)$, is conventionally assumed to be constant (Royle et al., 2011). Here, however, we model the distribution of $s_{i,t}$ as a function of time‐varying habitat covariates by discretizing the study area S into a grid of G pixels. Then, expected density in each grid pixel, $E\left(D_{g,t}\right)$ {g ϵ 1, 2, …, G}, varies across both space and time with a set of J covariates, $X_{j}$,

$$\log\left(E\left(D_{g,t}\right)\right)=\sum_{j=1}^{J}\beta_{j}X_{j,g,t},$$

and $s_{i,t}$ is modeled as a categorical random variable,

$$s_{i,t}∼\text{Categorical}\left(\pi_{Density,1,t}\ldots \pi_{Density,G,t}\right),$$

where

$$\pi_{Density,g,t}=E\left(D_{g,t}\right)/\sum_{g=1}^{G}E\left(D_{g,t}\right).$$

Here, $s_{i,t}$ references a pixel defined by its center coordinates (Linden et al., 2018). The open population model specifies total abundance in each primary period, $N_{t}$, while the density model specifies how those individuals are distributed across the study area; hence, there is no need for an intercept term in the density model, and $E\left(D_{g,t}\right)$ represents expected relative density. With a state–space formulation, realized absolute density, $R\left(D_{g,t}\right)$, can be derived as the number of activity centers in pixel g at time t divided by the area of the pixel.

**FIGURE 1:**
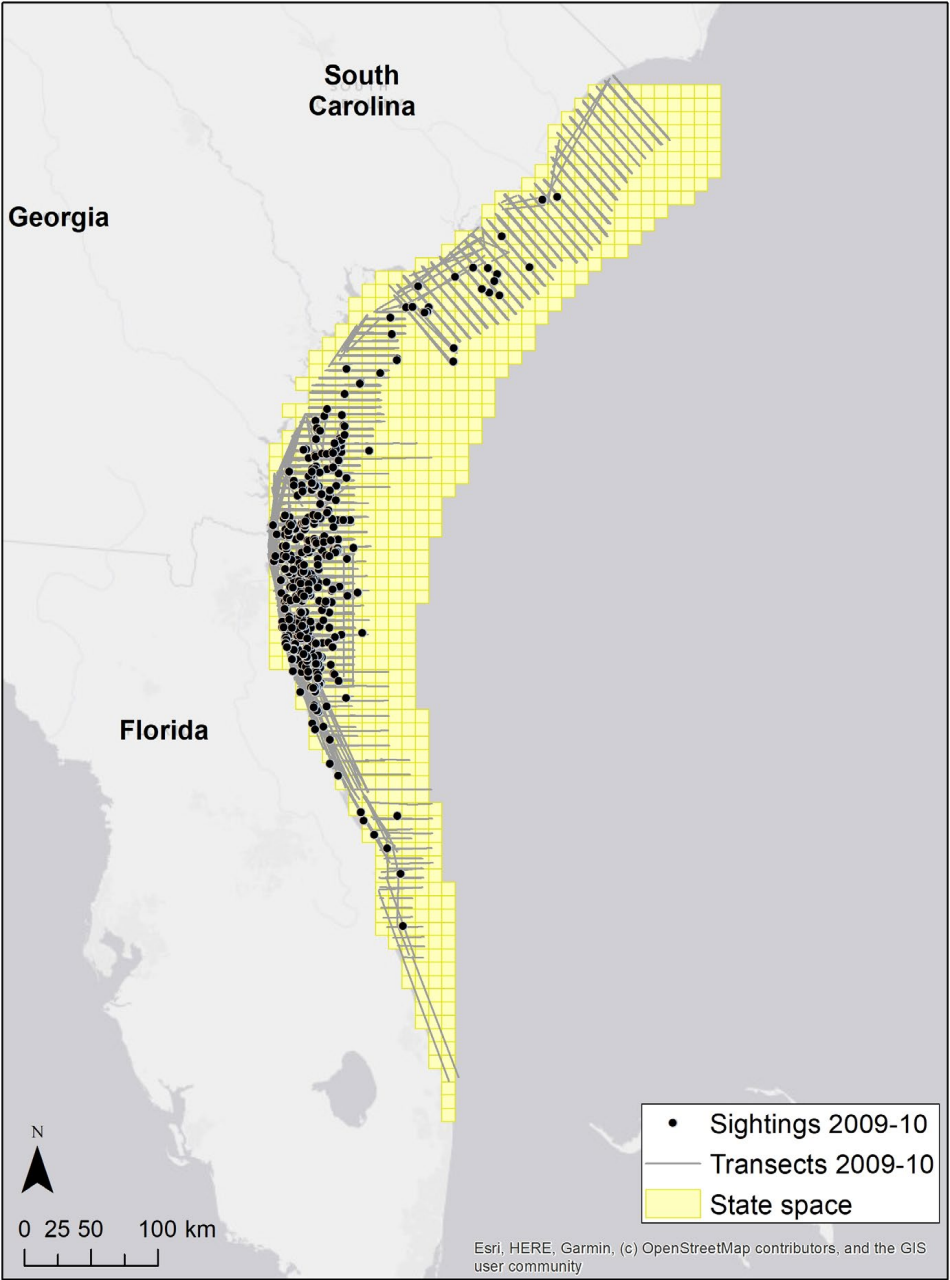
Study area for right whale analysis, discretized into 10 km × 10 km grid pixels, with survey transects and observed sightings from the 2009–2010 winter

We defined the right whale state–space region as water between 0 m and 70 m deep, between 2,960 km and 3,740 km north (NAD83, UTM zone 17N), and west of 800 km east, discretized into 10 km × 10 km pixels for a total of G = 772 pixels (Figure 1). Right whale density was modeled as a quadratic function of sea surface temperature ($sst_{g,t}$), wind speed ($ws_{g,t}$), and water depth ($depth_{g}$),

$$\log\left(E\left(D_{g,t}\right)\right)=\beta_{SST1}sst_{g,t}+\beta_{SST2}sst_{g,t}^{2}+\beta_{WS1}ws_{g,t}+\beta_{WS2}ws_{g,t}^{2}+\beta_{Depth1}depth_{g}+\beta_{Depth2}depth_{g}^{2}.$$

Sea surface temperature and water depth data were summarized following Gowan and Ortega‐Ortiz (2014), and wind speed was summarized from the Cross‐Calibrated Multi‐Platform wind vector analysis product (Wentz et al., 2015). Sea surface temperature and wind speed values were averaged within each pixel and each primary period; water depth values were averaged within each pixel but were constant across time. Pixels with missing data were interpolated as the mean of neighboring pixels within 15 km, and each variable was standardized to mean = 0 and standard deviation = 1.

### 2.4 Movement model

A movement, or space usage, model is used to model the probability, $\pi_{Movement,i,g,t}$, that individual i is in a given location (pixel g) on secondary sampling occasion $k_{t}\in K_{t}$. Following Royle et al. (2013), we modeled this movement probability as a function of $d_{i,g,t}$ (the distance between pixel g and $s_{i,t}$) and as a function of habitat covariates,

$$\text{log}\left(\mu_{i,g,t}\right)=‐\frac{1}{2\sigma_{\mathrm{move}}^{2}}d_{i,g,t}^{2}+\sum_{j=1}^{J}\beta_{j}X_{j,g,t},$$

$$\pi_{Movement,i,g,t}=\mu_{i,g,t}/\sum_{g=1}^{G}\mu_{i,g,t}.$$

Thus, if the movement scale parameter, $\sigma_{\mathrm{move}}$, is small, the inference is that individuals are less likely to move far from their activity centers. In contrast to standard spatial capture–recapture models, this movement model allows inference on resource selection functions (Royle et al., 2013), does not assume that space use is symmetric, and can be used to preclude estimated locations from occurring in unsuitable habitat (e.g., whales on land). In the right whale example, the habitat covariates and coefficients that influenced movement were the same as those that influenced density, and the discretized state–space for movement was the same as that for activity centers, but neither of these constraints is required (Linden et al., 2018).

Adapting the search‐encounter model, we permit animals to be detected anywhere in continuous space. Accordingly, the pixel containing an individual's location on sampling occasion $k_{t}$ is

$$U_{Pixel,i,k,t}∼\text{Categorical}\left(\pi_{Movement,i,1,t}\ldots \pi_{Movement,i,G,t}\right),$$

and we assume a uniform prior for the individual's exact location within that square pixel,

$$U_{x,i,k,t}∼\text{Uniform}\left(U_{xPixel,i,k,t}‐\frac{\mathrm{side}}{2},U_{xPixel,i,k,t}+\frac{\mathrm{side}}{2}\right),$$

$$U_{y,i,k,t}∼\text{Uniform}\left(U_{yPixel,i,k,t}‐\frac{\mathrm{side}}{2},U_{yPixel,i,k,t}+\frac{\mathrm{side}}{2}\right),$$

where side is the width of the pixel, and $U_{xPixel,i,k,t}$ and $U_{yPixel,i,k,t}$ are the *x*‐ and *y*‐coordinates of the pixel's center.

### 2.5 Detection model

Detection probability, $p_{i,k,t}$, is a function of $\min_{l\in L}\left(dist_{i,k,t,l}\right)$, the distance between the individual's location [$U_{x,i,k,t}$, $U_{y,i,k,t}$] and the closest point on the closest survey transect out of L total transects on secondary sampling occasion $k_{t}$. Distance between [$U_{x,i,k,t}$, $U_{y,i,k,t}$] and a survey transect, l, is calculated as

$$dist_{i,k,t,l}=\left\{\begin{array}{cc} \frac{\left|\left(y_{l,2}‐y_{l,1}\right)U_{x,i,k,t}‐\left(x_{l,2}‐x_{l,1}\right)U_{y,i,k,t}+x_{l,2}y_{l,1}+y_{l,2}x_{l,1}\right|}{\sqrt{{\left(x_{l,2}‐x_{l,1}\right)}^{2}+{\left(y_{l,2}‐y_{l,1}\right)}^{2}}}, & \text{if}\;x_{l,1}\leq \frac{U_{x,i,k,t}+m_{l}U_{y,i,k,t}‐m_{l}b_{l}}{m_{l}^{2}+1}\leq x_{l,2}\\ \min\left(\sqrt{{\left(x_{l,1}‐U_{x,i,k,t}\right)}^{2}+{\left(y_{l,1}‐U_{y,i,k,t}\right)}^{2}},\sqrt{{\left(x_{l,2}‐U_{x,i,k,t}\right)}^{2}+{\left(y_{l,2}‐U_{y,i,k,t}\right)}^{2}}\right), & \text{otherwise} \end{array},\right.$$

where [$x_{l,1},y_{l,1}$] is the westernmost point, [$x_{l,2},y_{l,2}$] is the easternmost point, $m_{l}$ is the slope, and $b_{l}$ is the *y*‐intercept of survey transect l (Ballantine & Jerbert, 1952). We provide R code in the Supporting Information for calculating $\min\left(\mathrm{dist}\right)$ when survey transects (or segments) are defined by the *x*‐ and *y*‐coordinates of their start and end points and are assumed to follow a straight line. The detection function may take the form of those commonly used in distance sampling (Buckland et al., 2001). For example, in our application and following Gowan and Ortega‐Ortiz (2014), we used a half‐normal detection function if the closest survey transect was flown by a Twin Otter airplane,

$$p_{i,k,t}=p_{0,hn}\exp\left(‐\frac{\min{\left(dist_{i,k,t,l}\right)}^{2}}{2\sigma_{\mathrm{hn}}^{2}}\right),$$

and we used a hazard‐rate detection function if the closest survey transect was flown by a Skymaster airplane

$$p_{i,k,t}=p_{0,hr}\left(1‐\exp\left(‐{\left(\frac{\min\left(dist_{i,k,t,l}\right)}{\sigma_{\mathrm{hr}}}\right)}^{‐b}\right)\right).$$

**FIGURE 2:**
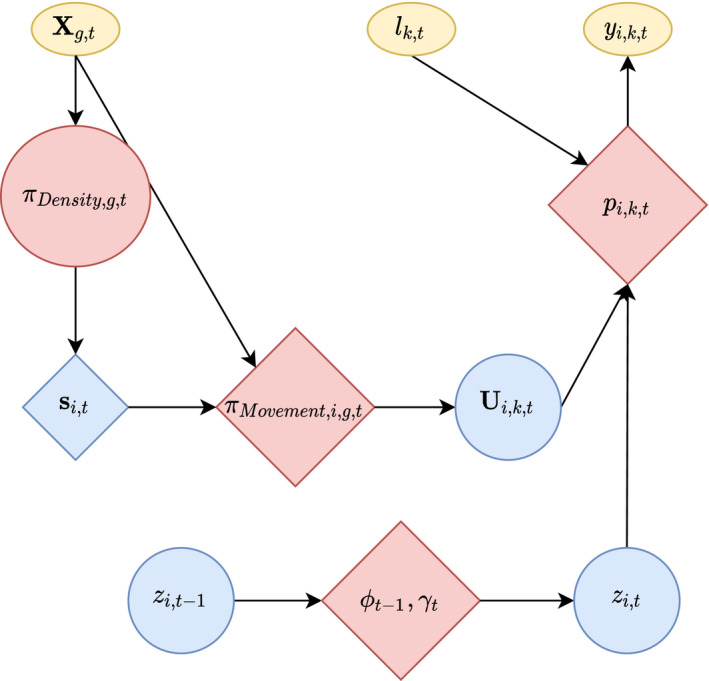
Diagram of model components, including observed data (boxes), latent state variables (circles), and estimated probability parameters (diamonds). The state variable $z_{i,t}$, indicating whether individual i was in the population (or study area) during primary sampling period t, is influenced by probabilities of survival (or persistence), ϕ, and recruitment (or arrival), γ. Spatiotemporal covariates, $X_{g,t}$, influence the density of activity centers, s, and an individual's location, $U_{i,k,t}$, on secondary sampling occasion k. Detection probability, p, influences whether an individual is detected, y, and is determined by the distance between the individual's location and the nearest survey transect, $l_{k,t}$

Note that, in distance sampling, $p_{0}$ (detection probability when $\min\left(\mathrm{dist}\right)$ = 0) is conventionally assumed to be 1, whereas, in spatial capture–recapture models, it is an estimable parameter. The observation model is

$$y_{i,k,t}∼\text{Bernoulli}\left(z_{i,t}p_{i,k,t}\right),$$

where $y_{i,k,t}=1$ if individual i was detected on secondary occasion $k_{t}$ and $y_{i,k,t}=0$ if individual i was not detected. The individual's location [$U_{x,i,k,t}$, $U_{y,i,k,t}$] is observed and supplied as data to the model when $y_{i,k,t}=1$ but is latent when $y_{i,k,t}=0$. Figure 2 illustrates how the observation model is related to input data and state variables from the other model components.

Details for the aerial surveys and photo‐identification data used in our right whale example are described elsewhere (Gowan & Ortega‐Ortiz, 2014; Krzystan et al., 2018). We excluded portions of surveys when the airplane was conducting a verification survey (i.e., responding to a reported whale sighting), circling a sighting, transiting between adjacent transects, or otherwise off watch. Longer transits (e.g., from the airport to the first survey transect) were retained if observers were on watch. Survey segments were defined by the start and end points of continuous portions of on‐watch effort and assumed to follow a straight line. While additional survey covariates may be included in the detection model, surveys were generally flown only during favorable weather conditions (e.g., visibility ≥3.7 km, Beaufort sea state ≤4).

Survey airplanes deviated from planned transects to photograph detected whales for individual identification and to collect whale locations. We excluded detections made during verification surveys or while otherwise off watch, and we excluded first‐year, dependent calves from the analysis. In this model, an individual may be detected once at most on each secondary sampling occasion. When an individual whale was detected more than once on the same survey day, we excluded detections made during transits or retained only the earliest detection made that day.

### 2.6 Group effects

When data are collected from different strata or groups, it is often desirable to combine the data into a single analysis in which some parameters are assumed constant across strata while other parameters vary. Such strata may be spatial or temporal replicates (Royle & Converse, 2014) or, as we illustrate, different demographic groups. This is relatively straightforward to implement by including the group index for V groups as a covariate for any component of the model. A key challenge with capture–recapture data is that group membership is not known for individuals that are not detected and it might not be known even for individuals that are detected (Genovart et al., 2012). To address this, we treat $group_{i}$, the group index for individual i, as data when it is known and as a latent variable when it is unknown (including for all M‐n individuals). We used a noninformative Dirichlet prior, specified with gamma hyperparameters (Kéry & Schaub, 2012), to model group index,

$$group_{i}∼\text{Categorical}\left(\pi_{Group,1}\ldots \pi_{Group,V}\right),$$

$$\pi_{Group,v}=a_{v}/\sum_{v=1}^{V}a_{v},$$

$$a_{v}∼\text{Gamma}\left(1,1\right).$$

In our application, we used V = 2 groups (calving females, all other demographic groups) and allowed arrival ($\beta_{\gamma }$), persistence ($\beta_{\phi }$), movement scale ($\sigma_{\mathrm{move}}$), and baseline detection ($p_{0}$, additive effect across survey platforms) to differ between these groups. Our a priori hypotheses were that calving females would arrive earlier and remain longer in the study area (Krzystan et al., 2018), move shorter distances, and spend more time near the surface (thus increasing their availability for detection) than the other group, but that the effects of habitat covariates on density and movement, and the effect of distance on detection, would be the same for both groups.

### 2.7 Implementation

We fit the model using the R (version 3.6.0) package nimble (version 0.7.1), a Markov chain Monte Carlo (MCMC) implementation of the BUGS language (NIMBLE Development Team, 2019). To improve computational efficiency, we used compiled functions in nimble (de Valpine et al., 2017) to calculate: $\pi_{Density,1:G,1:T}$ based on habitat covariates and sampled values for habitat coefficients; $\pi_{Movement,i,1:G,t}$ based on habitat covariates and sampled values for $s_{i,t}$ and habitat coefficients; and $p_{i,k,t}$ based on location and platform index of survey transects and sampled values for an individual's location [$U_{x,i,k,t}$, $U_{y,i,k,t}$] and detection function parameters. In the Supporting Information, we provide R code to simulate data and fit the model, as well as results from a simulation study.

For the right whale application, the coordinate system was scaled (1 unit distance = 10 km) to aid model convergence. One sighting of a group of 5 individuals was deemed likely an uncoded verification sighting ($\min\left(\mathrm{dist}\right)$ = 20.1 km, more than twice that of any other detection); this sighting was removed from the data to reduce its influence on the detection function (Buckland et al., 2001). Uniform priors were used for all arrival ($\beta_{\gamma }$), persistence ($\beta_{\phi }$), density ($\beta_{\mathrm{SST}},\beta_{\mathrm{WS}},\beta_{\mathrm{Depth}}$), movement ($\sigma_{\mathrm{move}}$), and detection ($p_{0},\sigma_{\text{hn}},\sigma_{\mathrm{hr}},b$) parameters. The data‐augmentation parameter M was initially set at 500 but was reduced to 350 after preliminary model runs suggested that it would reduce model runtime without affecting parameter estimates. We ran three MCMC chains of 12,000 iterations, discarding the first 6,000 as burn‐in. We present the mean of the posterior distribution for each parameter, with 2.5% and 97.5% quantiles as 95% Bayesian credible intervals. We assessed chain convergence using the Gelman–Rubin statistic, $\hat{R}$, and visual examination of the chains (Gelman et al., 2004). $\hat{R}$ values were <1.1, indicating convergence, for all parameters except the six habitat coefficients ($\hat{R}$ range: 1.1–2.1). Collinearity caused by including the squared values of each habitat covariate likely hindered convergence of these individual coefficients (Shacham & Brauner, 1997), but the functional relationships for each covariate were still estimated with reasonable precision, and simulations indicated that this artifact did not bias estimates of the functional relationships or other parameters.

## 3 RESULTS

**FIGURE 3:**
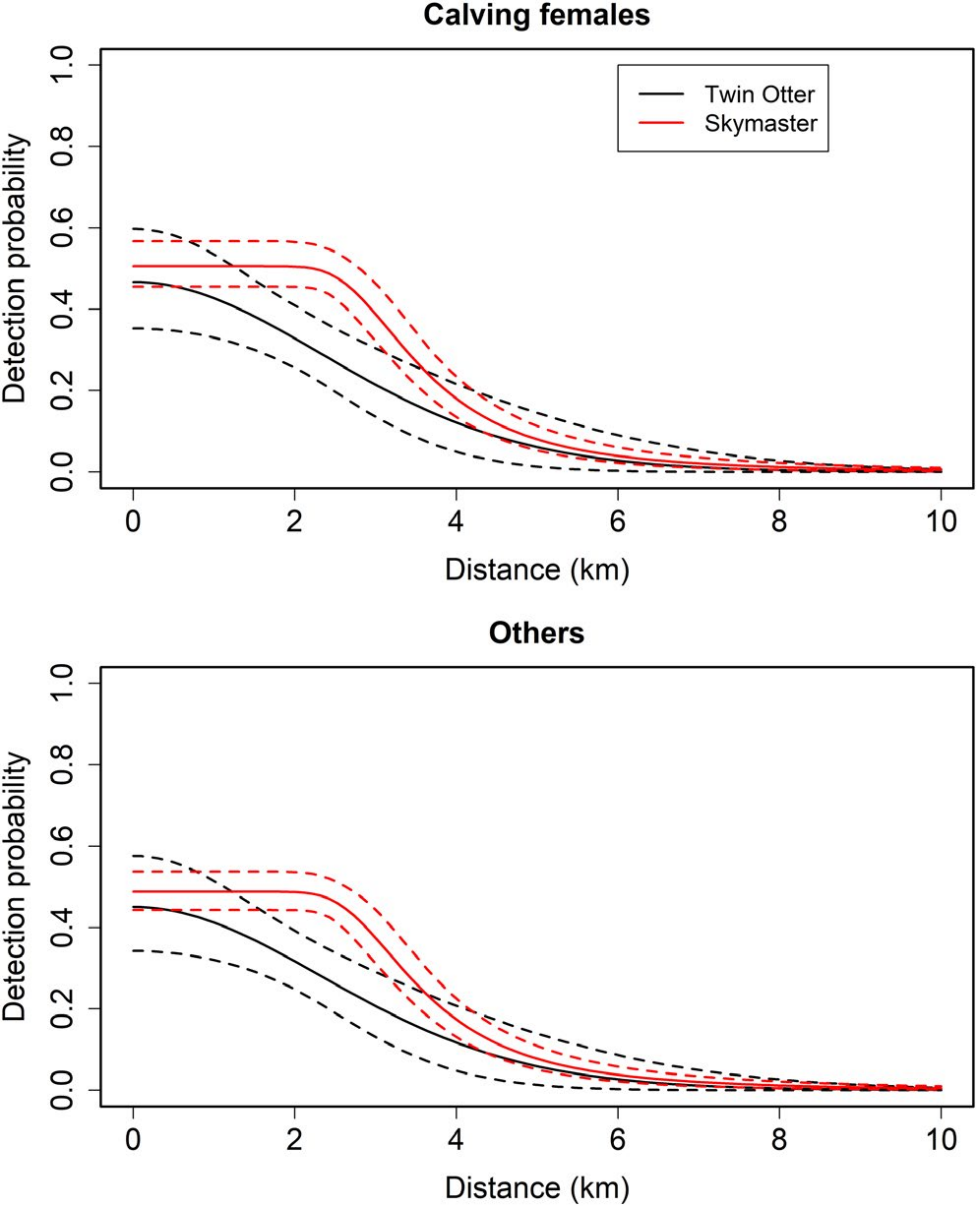
Estimated detection probability (with 95% credible intervals, dashed lines) as a function of distance for two survey platforms (Twin Otter and Skymaster aircraft) for calving female (top panel) and all other (bottom panel) right whales

**FIGURE 4:**
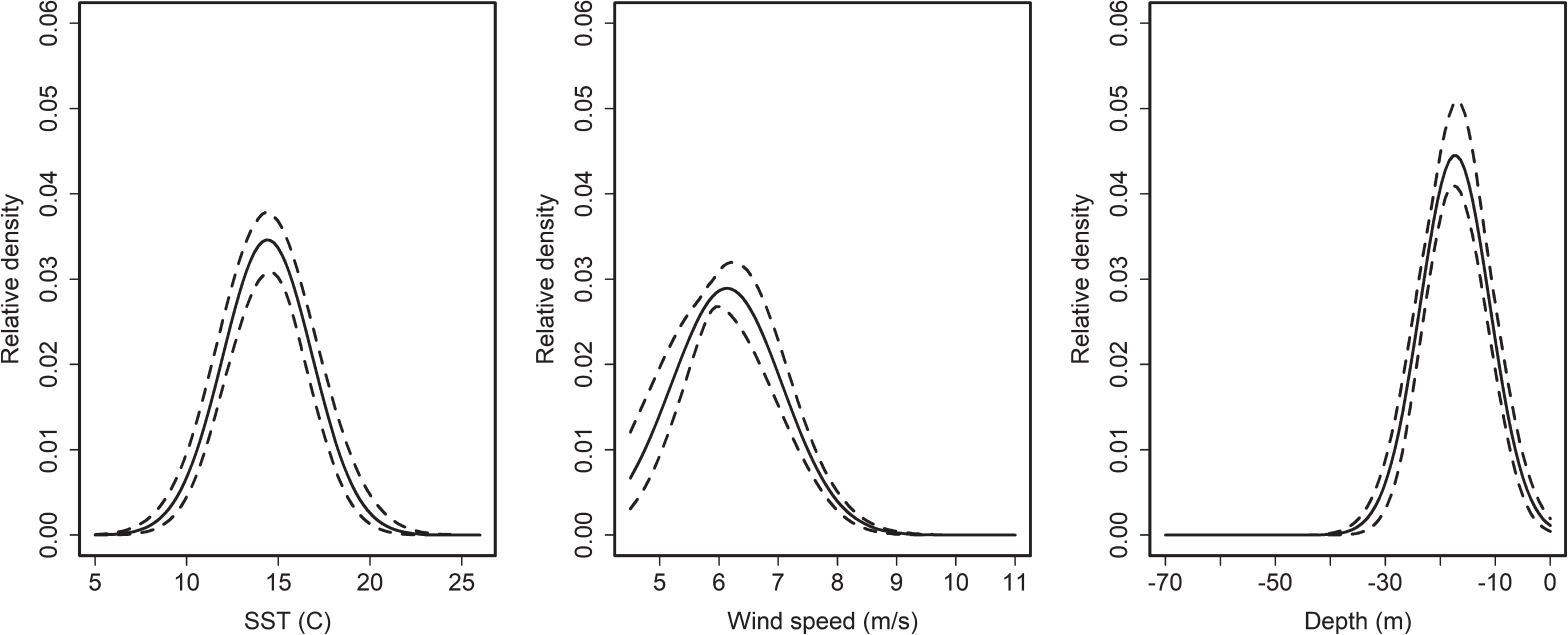
Expected relative right whale density (with 95% credible intervals, dashed lines) as a function of sea surface temperature (SST), wind speed, and water depth. In each panel, the other two covariates are held constant at their mean value

A total of 214 individual right whales (19 calving females and 195 others) were observed and retained for analysis during the winter of 2009–2010; the number of detections of each individual ranged from 1 to 17 for the entire winter and from 0 to 5 for within a primary period. The level of survey effort in each primary period ranged from 7 to 15 survey days (7,316 km to 26,333 km flown). Estimated detection functions were nearly identical across demographic groups, and $p_{0}$ was similar across survey platforms (Figure 3). Calving females tended to move shorter distances within the study area than did other right whales, although the distance from activity center had little effect on space use for both groups, and credible intervals overlapped (calving females: $\sigma_{\mathrm{move}}$ = 7.97, 95% credible interval = [6.13, 13.36]; others: $\sigma_{\mathrm{move}}$ = 11.45 [9.78, 13.54]). Expected density and space use probabilities peaked at around 12.6–16.0°C sea surface temperature, 5–7 m/s wind speed, and 10.6–24.0 m water depth (Figure 4).

**FIGURE 5:**
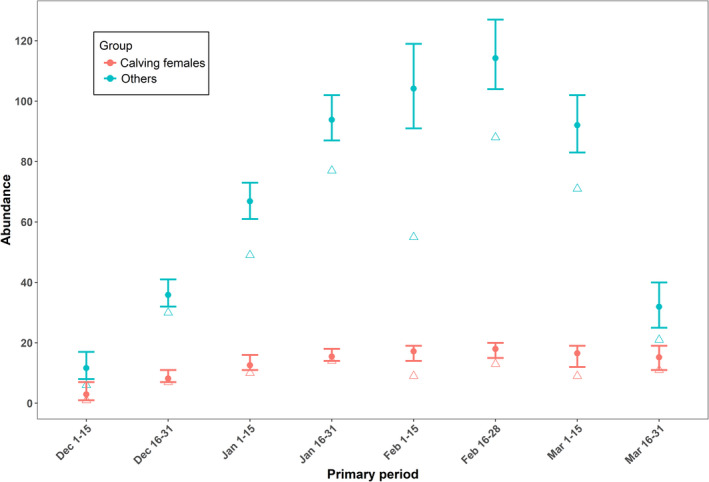
Estimated abundance (circles; with 95% credible intervals) and observed number of individuals (open triangles; i.e., uncorrected count) by primary period for calving female and all other right whales

**FIGURE 6:**
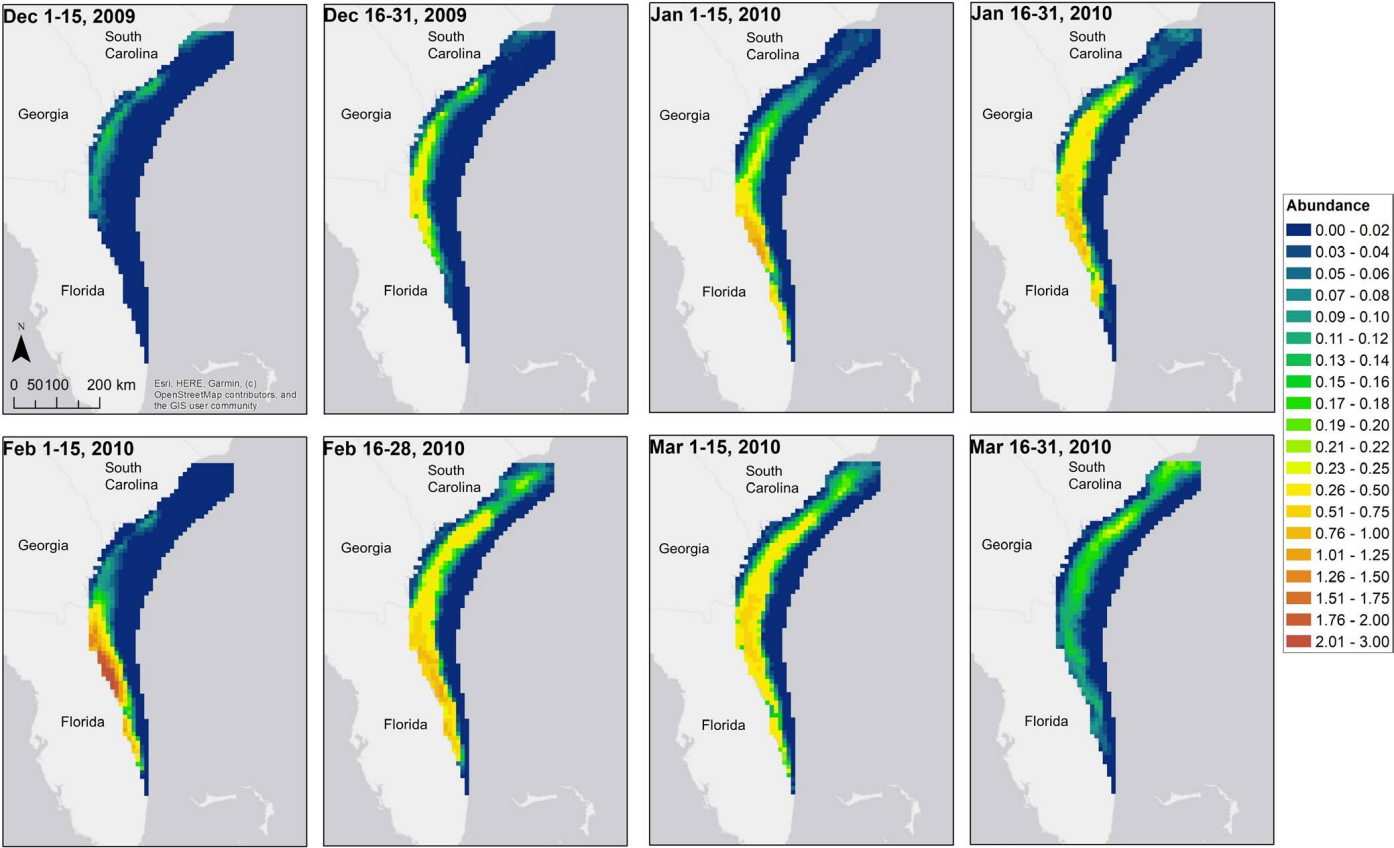
Estimated density (i.e., abundance in 10 km × 10 km grid pixels) of right whales by primary period

Nineteen [19, 21] calving females and 217 [207, 227] other right whales were estimated to have been in the study area at some point during the winter. Calving females were most likely to arrive in late December or early January, whereas other whales were more likely to arrive in late February and early March (Figure S3). Calving females were very likely to remain in the study area through at least early March, while other groups were more likely to depart (Figure S4). For both groups, estimated abundance was highest in February (Figure 5). Realized absolute density estimates reflected an increasing number of whales in the study area from early December through late February, followed by decreasing density in March (Figures 5 and 6). Areas of highest density were located in the northern portion of the study area in the beginning of winter (when sea surface temperatures were highest), shifted south in the middle of the winter (when temperatures were lowest), and then shifted back north at the end of the winter (Figure 6).

## 4 DISCUSSION

Most abundance models for line‐transect surveys are designed to estimate abundance within an area at discrete points in time. However, the open spatial capture–recapture model presented here permits inference on factors that influence spatial and temporal variation in density, movement, and population dynamics within a single framework. This model offers several advantages over other methods for estimating abundance. For example, in conventional distance sampling, $p_{0}$ is assumed to be 1, or an ad hoc correction factor is used based on ancillary information, such as diving behavior for aquatic species (Roberts et al., 2016). In contrast, the model presented here estimates $p_{0}$ directly using information from the recapture of identified individuals, similar to capture–recapture distance sampling that relies on multiple observers (Borchers et al., 1998). In our case study, we estimated variation in $p_{0}$ due to behavioral differences across demographic groups as well as survey platform configurations (Figure 3). Moreover, because individuals are tracked across survey occasions, our model provides improved inference on population processes such as recruitment, mortality, and residency that drive temporal variation in abundance. We believe this aspect can be particularly useful in quantifying cumulative impacts based on the amount of time an individual is exposed to stressors in an area (Pirotta et al., 2018). For example, we estimated that most calving female right whales were present in the study area from at least early January through March, indicating that they are exposed to threats such as ship traffic and noise impacts in this region throughout the entire winter.

In contrast to conventional capture–recapture methods, the area of inference is explicitly defined in spatial capture–recapture models (Borchers & Marques, 2017). This distinction may be especially important when there are changes over time in the survey extent or spatial patterns of abundance (e.g., due to movement of mobile species). Additionally, variation in density across space can be inferred and used to inform management decisions, such as the placement of protection zones (Udell et al., 2019) and the design of future monitoring surveys. Modeling heterogeneity in the spatial distribution of individual activity centers and movements allowed us to account for the effect of proximity to survey transects on an individual's detection probability. Ignoring this heterogeneity in detection probabilities is known to bias abundance estimates in both conventional and spatial capture–recapture models (Royle et al., 2013). Finally, while most spatial capture–recapture models have been developed for detectors arranged in a fixed array, our model can be applied to data collected from continuous line transects, without the need to approximate those lines as points.

Our case study was limited to only a portion of the North Atlantic right whale's range, but the results are relevant to understanding reproduction (via the number of calving females) and migration patterns, as well as to quantifying risk from human activities in the area. For example, frameworks used to predict ship‐strike mortality risk for whales require accurate estimates of whale density and residency as inputs (Crum et al., 2019; van der Hoop et al., 2012). Estimated patterns of right whale spatial distribution and residency in this study were consistent with previous work in the region (Gowan & Ortega‐Ortiz, 2014; Krzystan et al., 2018; Roberts et al., 2016), but our model integrates these components in a single analysis that provides estimates of absolute density while relaxing assumptions about $p_{0}$. Additionally, our framework includes an explicit model for individual movement, which highlighted the large scale of right whale movements and the importance of accounting for environmental factors. Unlike most spatial capture–recapture models, which implicitly assume that space use within an individual's home range is symmetric and stationary (e.g., Royle et al., 2011), we modeled space use as a function of habitat covariates, which will be more appropriate for species that seek patchily distributed resources (Curtice et al., 2015; Royle et al., 2013).

Right whale survey data from additional winters could be included within the same framework to examine interannual variation in migration dynamics and density, while assuming that some parameters related to detection and movement may be constant across years. Multiple years of data could be estimated jointly in a single analysis or in a sequential manner using posterior estimates from previous seasons as priors. The methodology can also be applied to right whale surveys in other regions that use photo‐identification and record the locations of sightings and survey transects, with the potential for estimating density throughout the species' range and population dynamics across years. However, different habitat covariates are likely needed to model density in different regions (e.g., calving grounds versus feeding grounds), and using such covariates to extrapolate density to areas with sparse survey effort should be done cautiously (Wenger & Olden, 2012).

We believe there is great potential to apply our spatial capture–recapture model, or modifications thereof, to other monitoring programs, but we acknowledge the trade‐off between survey costs and level of inference gained. For example, the identification of individuals typically results in increased survey time or costs, although numerous methods for doing so exist (Hammond, 2009), and it is expected to provide increased information about $p_{0}$ and population dynamics. Beyond visual and genetic identification methods, the application of spatial capture–recapture models for passive acoustic data has received some attention (Marques et al., 2012), but challenges associated with identifying individuals from vocalizations and accounting for nonvocalizing individuals remain (Stevenson et al., 2015). If detection distances are not available, alternative detection models can be used (e.g., Russell et al., 2012) while still incorporating the population dynamics, density, and movement model components presented here. Naturally, model complexity and parameter estimability will be limited by the number of detections, as well as the frequency, timing, and spatial extent of surveys. Previous work has highlighted the utility of integrated population models when large‐scale capture–recapture studies are not feasible. For example, to estimate both abundance and demographic parameters, Chandler et al. (2018) and Schmidt and Robison (2020) combined distance sampling data with capture–recapture data, which were collected through separate studies and considered conditionally independent. Alternatively, our framework combines individual identification and distance data that are collected simultaneously during a single study, and it allows for explicitly modeling individual space use and its influence on detection, including the effects of habitat covariates on space use when assumptions for symmetric home ranges are not met.

Several extensions to our model may be warranted. First, our detection model did not account for the effect of group size or the potential nonindependence of temporarily associated individuals. Despite our expectation that calving females spend more time near the water surface, estimated detection probabilities were similar across demographic groups. This may be due in part to the tendency of other whales to aggregate in surface active groups, increasing their availability for detection. One potential solution to account for group size is to enumerate the number of other individuals within a specified distance of [$U_{x,i,k,t}$, $U_{y,i,k,t}$] for each MCMC iteration, effectively treating group size as a latent variable, and using this as a covariate in the detection function. While nonindependent detections induce negligible bias in spatial capture–recapture models, especially when group sizes are small, they may inflate precision (Bischof et al., 2020). Future work should consider explicitly modeling grouping behavior and an observation process where both groups and individuals within a detected group may go undetected (Clement et al., 2017). Additionally, we assumed that detection and movement parameters were constant throughout the winter, yet calving females typically do not give birth until sometime after they arrive in the study area. A multistate model could be developed to account for behavioral differences between pregnant females and females with a calf. Moreover, some individuals that we classified into the other group may have in fact been calving females whose calves were never detected, so group index could be regarded as an unknown parameter even for individuals that are detected.

Including activity center, $s_{i}$, as a latent variable is a primary component of all spatial capture–recapture models. However, estimates for $\sigma_{\mathrm{move}}$ were relatively large in our case study, indicating that whales had limited fidelity to these estimated activity centers. Rather than estimating $s_{i}$, an alternative approach might estimate an individual's location when it is first recruited (either with a uniform prior or as a function of habitat covariates), then extend our movement model to estimate subsequent locations with a correlated random walk. In this scenario, $d_{i,g,t}$ would refer to the distance from an individual's previous location rather than its activity center, and inferences on density would relate to an individual's true location on each occasion rather than to a stationary activity center. This approach may be more relevant for highly mobile species with limited site fidelity, and it may be useful in distinguishing true mortality from dispersal from the survey area (Schaub & Royle, 2014). In any case, available telemetry data can be integrated to inform movement parameters and improve resulting estimates of density (Royle et al., 2013).

Modifying our model based on study species, parameters of interest, and available data is relatively straightforward due to the availability of software for specifying customized Bayesian models (de Valpine et al., 2017). However, model runtimes using MCMC can be prohibitive in some situations, so we encourage the development of maximum‐likelihood approaches (Glennie et al., 2019) and other solutions to improve computational efficiency while retaining ease of customization. Such developments should make open spatial capture–recapture models more accessible and amenable to model selection in order to improve inferences on abundance, space use, and population dynamics from monitoring data.

## CONFLICT OF INTEREST

The authors have no conflicts of interest to declare.

## AUTHOR CONTRIBUTIONS

**Timothy A. Gowan:** Conceptualization (lead); data curation (lead); formal analysis (lead); software (lead); writing‐original draft (lead); writing‐review & editing (equal). **Nathan J. Crum:** Conceptualization (supporting); data curation (supporting); software (supporting); writing‐review & editing (equal). **Jason J. Roberts:** Data curation (supporting); formal analysis (supporting); writing‐review & editing (equal).

## Supporting information

Supplementary Material

Fig S1‐S4

## Data Availability

The data used for the right whale analysis are available on the Dryad Digital Repository: https://doi.org/10.5061/dryad.stqjq2c35.

## References

[ece37566-bib-0001] Ballantine, J. P. , & Jerbert, A. R. (1952). Distance from a line, or plane, to a point. The American Mathematical Monthly, 59(4), 242. 10.2307/2306514

[ece37566-bib-0002] Bischof, R. , Dupont, P. , Milleret, C. , Chipperfield, J. , & Royle, J. A. (2020). Consequences of ignoring group association in spatial capture–recapture analysis. Wildlife Biology, 2020(1), 1–10. 10.2981/wlb.00649

[ece37566-bib-0003] Borchers, D. L. , & Marques, T. A. (2017). From distance sampling to spatial capture–recapture. AStA Advances in Statistical Analysis, 101(4), 475–494. 10.1007/s10182-016-0287-7

[ece37566-bib-0004] Borchers, D. L. , Stevenson, B. C. , Kidney, D. , Thomas, L. , & Marques, T. A. (2015). A unifying model for capture–recapture and distance sampling surveys of wildlife populations. Journal of the American Statistical Association, 110(509), 195–204. 10.1080/01621459.2014.893884 26063947 PMC4440664

[ece37566-bib-0005] Borchers, D. L. , Zucchini, W. , & Fewster, R. M. (1998). Mark‐recapture models for line transect surveys. Biometrics, 54(4), 1207. 10.2307/2533651

[ece37566-bib-0006] Buckland, S. T. , Anderson, D. R. , Burnham, K. P. , Laake, J. L. , Borchers, D. L. , & Thomas, L. (2001). Introduction to distance sampling: Estimating abundance of biological populations. Oxford University Press.

[ece37566-bib-0007] Chandler, R. B. , Hepinstall‐Cymerman, J. , Merker, S. , Abernathy‐Conners, H. , & Cooper, R. J. (2018). Characterizing spatio‐temporal variation in survival and recruitment with integrated population models. The Auk, 135(3), 409–426. 10.1642/AUK-17-181.1

[ece37566-bib-0008] Clement, M. J. , Converse, S. J. , & Royle, J. A. (2017). Accounting for imperfect detection of groups and individuals when estimating abundance. Ecology and Evolution, 7(18), 7304–7310. 10.1002/ece3.3284 28944018 PMC5606903

[ece37566-bib-0009] Constantine, R. , Jackson, J. A. , Steel, D. , Baker, C. S. , Brooks, L. , Burns, D. , Clapham, P. , Hauser, N. , Madon, B. , Mattila, D. , Oremus, M. , Poole, M. , Robbins, J. , Thompson, K. , & Garrigue, C. (2012). Abundance of humpback whales in Oceania using photo‐identification and microsatellite genotyping. Marine Ecology Progress Series, 453, 249–261. 10.3354/meps09613

[ece37566-bib-0010] Crum, N. , Gowan, T. , Krzystan, A. , & Martin, J. (2019). Quantifying risk of whale–vessel collisions across space, time, and management policies. Ecosphere, 10(4), e02713. 10.1002/ecs2.2713

[ece37566-bib-0011] Curtice, C. , Johnston, D. W. , Ducklow, H. , Gales, N. , Halpin, P. N. , & Friedlaender, A. S. (2015). Modeling the spatial and temporal dynamics of foraging movements of humpback whales (*Megaptera novaeangliae*) in the Western Antarctic Peninsula. Movement. Ecology, 3(1), 1–9. 10.1186/s40462-015-0041-x 26034604 PMC4450492

[ece37566-bib-0054] Dail, D. , & Madsen, L. (2011). Models for estimating abundance from repeated counts of an open metapopulation. Biometrics, 67(2), 577–587. 10.1111/j.1541-0420.2010.01465.x 20662829

[ece37566-bib-0012] de Valpine, P. , Turek, D. , Paciorek, C. J. , Anderson‐Bergman, C. , Lang, D. T. , & Bodik, R. (2017). Programming with models: Writing statistical algorithms for general model structures with NIMBLE. Journal of Computational and Graphical Statistics, 26(2), 403–413. 10.1080/10618600.2016.1172487

[ece37566-bib-0013] Farmer, N. A. , Gowan, T. A. , Powell, J. R. , & Zoodsma, B. J. (2016). Evaluation of alternatives to winter closure of Black Sea Bass pot gear: Projected impacts on catch and risk of entanglement with North Atlantic right whales *Eubalaena glacialis* . Marine and Coastal Fisheries, 8(1), 202–221. 10.1080/19425120.2016.1146181

[ece37566-bib-0014] Gardner, B. , Reppucci, J. , Lucherini, M. , & Royle, J. A. (2010). Spatially explicit inference for open populations: Estimating demographic parameters from camera‐trap studies. Ecology, 91(11), 3376–3383. 10.1890/09-0804.1 21141198

[ece37566-bib-0015] Gardner, B. , Sollmann, R. , Kumar, N. S. , Jathanna, D. , & Karanth, K. U. (2018). State space and movement specification in open population spatial capture‐recapture models. Ecology and Evolution, 8(20), 10336–10344. 10.1002/ece3.4509 30397470 PMC6206188

[ece37566-bib-0016] Gelman, A. , Carlin, J. B. , Stern, H. S. , & Rubin, D. B. (2004). Bayesian data analysis (2nd ed.). CRC Press.

[ece37566-bib-0017] Genovart, M. , Pradel, R. , & Oro, D. (2012). Exploiting uncertain ecological fieldwork data with multi‐event capture‐recapture modelling: An example with bird sex assignment. Journal of Animal Ecology, 81(5), 970–977. 10.1111/j.1365-2656.2012.01991.x 22548508

[ece37566-bib-0018] Glennie, R. , Borchers, D. L. , Murchie, M. , Harmsen, B. J. , & Foster, R. J. (2019). Open population maximum likelihood spatial capture‐recapture. Biometrics, 75(4), 1345–1355. 10.1111/biom.13078 31045249

[ece37566-bib-0019] Gowan, T. A. , & Ortega‐Ortiz, J. G. (2014). Wintering habitat model for the North Atlantic right whale (*Eubalaena glacialis*) in the southeastern United States. PLoS One, 9(4), e95126. 10.1371/journal.pone.0095126 24740091 PMC3989274

[ece37566-bib-0020] Hammond, P. S. (2009). Mark‐Recapture. In W. Perrin , B. Würsig , & J. G. M. Thewissen (Eds.), Encyclopedia of marine mammals (pp. 705–709). Academic Press. 10.1016/B978-0-12-373553-9.00163-2

[ece37566-bib-0021] Kéry, M. , & Royle, J. (2020). Applied hierarchical modeling in ecology: Analysis of distribution, abundance and species richness in R and BUGS: Dynamic and advanced models (1st ed., Vol. 2). Elsevier.

[ece37566-bib-0022] Kéry, M. , & Schaub, M. (2012). Bayesian population analysis using WinBUGS: A hierarchical perspective (1st ed.). Academic Press.

[ece37566-bib-0023] Krzystan, A. M. , Gowan, T. A. , Kendall, W. L. , Martin, J. , Ortega‐Ortiz, J. G. , Jackson, K. , Knowlton, A. R. , Naessig, P. , Zani, M. , Schulte, D. W. , & Taylor, C. R. (2018). Characterizing residence patterns of North Atlantic right whales in the southeastern USA with a multistate open robust design model. Endangered Species Research, 36, 279–295. 10.3354/esr00902

[ece37566-bib-0024] Linden, D. W. , Sirén, A. P. K. , & Pekins, P. J. (2018). Integrating telemetry data into spatial capture–recapture modifies inferences on multi‐scale resource selection. Ecosphere, 9(4), 1–14. 10.1002/ecs2.2203

[ece37566-bib-0025] Lyons, J. E. , Kendall, W. L. , Royle, J. A. , Converse, S. J. , Andres, B. A. , & Buchanan, J. B. (2016). Population size and stopover duration estimation using mark‐resight data and Bayesian analysis of a superpopulation model. Biometrics, 72(1), 262–271. 10.1111/biom.12393 26348116

[ece37566-bib-0026] Marques, T. A. , Thomas, L. , Martin, S. W. , Mellinger, D. K. , Jarvis, S. , Morrissey, R. P. , Ciminello, C.‐A. , & DiMarzio, N. (2012). Spatially explicit capture–recapture methods to estimate minke whale density from data collected at bottom‐mounted hydrophones. Journal of Ornithology, 152(S2), 445–455. 10.1007/s10336-010-0535-7

[ece37566-bib-0027] Miller, D. L. , Burt, M. L. , Rexstad, E. A. , & Thomas, L. (2013). Spatial models for distance sampling data: Recent developments and future directions. Methods in Ecology and Evolution, 4(11), 1001–1010. 10.1111/2041-210X.12105

[ece37566-bib-0028] Moore, J. E. , & Barlow, J. (2011). Bayesian state‐space model of fin whale abundance trends from a 1991–2008 time series of line‐transect surveys in the California Current. Journal of Applied Ecology, 48(5), 1195–1205. 10.1111/j.1365-2664.2011.02018.x

[ece37566-bib-0029] NIMBLE Development Team (2019). NIMBLE: An R package for programming with BUGS models, version 0.7‐1. http://r‐nimble.org

[ece37566-bib-0030] Pirotta, E. , Booth, C. G. , Costa, D. P. , Fleishman, E. , Kraus, S. D. , Lusseau, D. , Moretti, D. , New, L. F. , Schick, R. S. , Schwarz, L. K. , Simmons, S. E. , Thomas, L. , Tyack, P. L. , Weise, M. J. , Wells, R. S. , & Harwood, J. (2018). Understanding the population consequences of disturbance. Ecology and Evolution, 8(19), 9934–9946. 10.1002/ece3.4458 30386587 PMC6202709

[ece37566-bib-0031] Pollock, K. H. (1982). A capture‐recapture design robust to unequal probability of capture. The Journal of Wildlife Management, 46(3), 752. 10.2307/3808568

[ece37566-bib-0032] Roberts, J. J. , Best, B. D. , Mannocci, L. , Fujioka, E. I. , Halpin, P. N. , Palka, D. L. , Garrison, L. P. , Mullin, K. D. , Cole, T. V. N. , Khan, C. B. , McLellan, W. A. , Pabst, D. A. , & Lockhart, G. G. (2016). Habitat‐based cetacean density models for the U.S. Atlantic and Gulf of Mexico. Scientific Reports, 6(1), 1–12. 10.1038/srep22615 26936335 PMC4776172

[ece37566-bib-0033] Royle, J. A. , Chandler, R. B. , Sun, C. C. , & Fuller, A. K. (2013). Integrating resource selection information with spatial capture‐recapture. Methods in Ecology and Evolution, 4(6), 520–530. 10.1111/2041-210X.12039

[ece37566-bib-0034] Royle, J. A. , & Converse, S. J. (2014). Hierarchical spatial capture‐recapture models: Modelling population density in stratified populations. Methods in Ecology and Evolution, 5(1), 37–43. 10.1111/2041-210X.12135

[ece37566-bib-0035] Royle, J. A. , & Dorazio, R. M. (2009). Hierarchical modeling and inference in ecology: The analysis of data from populations, metapopulations and communities (Reprint). Academic Press.

[ece37566-bib-0036] Royle, J. A. , Fuller, A. K. , & Sutherland, C. (2018). Unifying population and landscape ecology with spatial capture‐recapture. Ecography, 41(3), 444–456. 10.1111/ecog.03170

[ece37566-bib-0037] Royle, J. A. , Kéry, M. , & Guélat, J. (2011). Spatial capture‐recapture models for search‐encounter data. Methods in Ecology and Evolution, 2(6), 602–611. 10.1111/j.2041-210X.2011.00116.x

[ece37566-bib-0038] Russell, R. E. , Royle, J. A. , Desimone, R. , Schwartz, M. K. , Edwards, V. L. , Pilgrim, K. P. , & Mckelvey, K. S. (2012). Estimating abundance of mountain lions from unstructured spatial sampling. The Journal of Wildlife Management, 76(8), 1551–1561. 10.1002/jwmg.412

[ece37566-bib-0039] Schaub, M. , & Royle, J. A. (2014). Estimating true instead of apparent survival using spatial Cormack‐Jolly‐Seber models. Methods in Ecology and Evolution, 5(12), 1316–1326. 10.1111/2041-210X.12134

[ece37566-bib-0040] Schmidt, J. H. , & Rattenbury, K. L. (2018). An open‐population distance sampling framework for assessing population dynamics in group‐dwelling species. Methods in Ecology and Evolution, 9(4), 936–945. 10.1111/2041-210X.12932

[ece37566-bib-0041] Schmidt, J. H. , & Robison, H. L. (2020). Using distance sampling‐based integrated population models to identify key demographic parameters. The Journal of Wildlife Management, 84(2), 372–381. 10.1002/jwmg.21805

[ece37566-bib-0042] Schwarz, C. J. , & Arnason, A. N. (1996). A general methodology for the analysis of capture‐recapture experiments in open populations. Biometrics, 52(3), 860. 10.2307/2533048

[ece37566-bib-0043] Shacham, M. , & Brauner, N. (1997). Minimizing the effects of collinearity in polynomial regression. Industrial & Engineering Chemistry Research, 36(10), 4405–4412. 10.1021/ie970236k

[ece37566-bib-0044] Smout, S. , King, R. , & Pomeroy, P. (2011). Estimating demographic parameters for capture–recapture data in the presence of multiple mark types. Environmental and Ecological Statistics, 18(2), 331–347. 10.1007/s10651-010-0135-y

[ece37566-bib-0045] Sollmann, R. , Gardner, B. , Chandler, R. B. , Royle, J. A. , & Sillett, T. S. (2015). An open‐population hierarchical distance sampling model. Ecology, 96(2), 325–331. 10.1890/14-1625.1 26240853

[ece37566-bib-0046] Stevenson, B. C. , Borchers, D. L. , Altwegg, R. , Swift, R. J. , Gillespie, D. M. , & Measey, G. J. (2015). A general framework for animal density estimation from acoustic detections across a fixed microphone array. Methods in Ecology and Evolution, 6(1), 38–48. 10.1111/2041-210X.12291

[ece37566-bib-0047] Taylor, C. M. , & Hastings, A. (2004). Finding optimal control strategies for invasive species: A density‐structured model for *Spartina alterniflora* . Journal of Applied Ecology, 41(6), 1049–1057. 10.1111/j.0021-8901.2004.00979.x

[ece37566-bib-0048] Udell, B. J. , Martin, J. , Fletcher, R. J. , Bonneau, M. , Edwards, H. H. , Gowan, T. A. , Hardy, S. K. , Gurarie, E. , Calleson, C. S. , & Deutsch, C. J. (2019). Integrating encounter theory with decision analysis to evaluate collision risk and determine optimal protection zones for wildlife. Journal of Applied Ecology, 56(5), 1050–1062. 10.1111/1365-2664.13290

[ece37566-bib-0049] van der Hoop, J. M. , Vanderlaan, A. S. M. , & Taggart, C. T. (2012). Absolute probability estimates of lethal vessel strikes to North Atlantic right whales in Roseway Basin, Scotian Shelf. Ecological Applications, 22(7), 2021–2033. 10.1890/11-1841.1 23210317

[ece37566-bib-0050] Wenger, S. J. , & Olden, J. D. (2012). Assessing transferability of ecological models: An underappreciated aspect of statistical validation. Methods in Ecology and Evolution, 3(2), 260–267. 10.1111/j.2041-210X.2011.00170.x

[ece37566-bib-0051] Wentz, F. J. , Scott, J. , Hoffman, R. , Leidner, M. , Atlas, R. , & Ardizzone, J. (2015). Remote Sensing Systems Cross‐Calibrated Multi‐Platform (CCMP) 6‐hourly ocean vector wind analysis product on 0.25 deg grid, Version 2.0. Remote Sensing Systems. www.remss.com/measurements/ccmp

[ece37566-bib-0052] Williams, B. K. , Nichols, J. D. , & Conroy, M. J. (2002). Analysis and management of animal populations: Modeling, estimation, and decision making. Academic Press.

[ece37566-bib-0053] Zipkin, E. F. , Sillett, T. S. , Grant, E. H. C. , Chandler, R. B. , & Royle, J. A. (2014). Inferences about population dynamics from count data using multistate models: A comparison to capture–recapture approaches. Ecology and Evolution, 4(4), 417–426. 10.1002/ece3.942 24634726 PMC3936388

