# Supplementary material for: An open spatial capture–recapture model for estimating density, movement, and population dynamics from line‐transect surveys
Source: Ecol Evol. 2021 May 3;11(12):7354–65. doi: 10.1002/ece3.7566 (PMC8216936; doi:10.1002/ece3.7566)
Supplement: Supplementary file 2 — Fig S1‐S4 [file ECE3-11-7354-s001.docx]

**Supporting Information**

We conducted a simulation study to evaluate the performance of our open spatial capture–recapture model. We simulated 100 datasets and compared the model estimates to the true values used to simulate data. Each dataset had $T$ = 8 primary periods with $K$ = 5 secondary occasions within each primary period. The maximum number of individuals in the population, $M$, was set at 100 in the simulations and at 120 for the model-fitting. As in the right whale application, recruitment and survival probabilities were a quadratic function and linear function, respectively, of time. The state–space region was 5 units × 6 units discretized into 0.5 units × 0.5 units pixels ($G$ = 120). Density and movement were a quadratic function ($\beta_{1}$ = 0, $\beta_{2}$ = -1.2) of a spatial covariate that varied across primary periods and simulations. We set $\sigma_{move}$ = 1. Four horizontal transect lines (3 units length, spaced 1 unit apart) were surveyed on each secondary occasion. Detection probability was a half-normal function with $p_{0}$ = 0.8 and $\sigma_{hn}^{2}$ = 0.36. We fit each dataset using a single MCMC chain of 1,500 iterations, discarding the first 500 as burn-in. We calculated bias as the difference between the mean of the posterior distribution estimated from each dataset and the true simulating value. Because recruitment and survival were stochastic, true values for $R_{t}$ and $N_{t}$ varied across simulations but were tracked to calculate bias.

There was no apparent bias in the leading parameters of the open spatial capture–recapture model (Figure S1). Bias was also negligible for abundance and number of recruits in each primary period, although abundance was slightly overestimated (mean bias = 0.54 individuals) and number of recruits was slightly underestimated (mean bias = -0.55 individuals), especially during the middle of the study when abundance was highest (Figure S2).


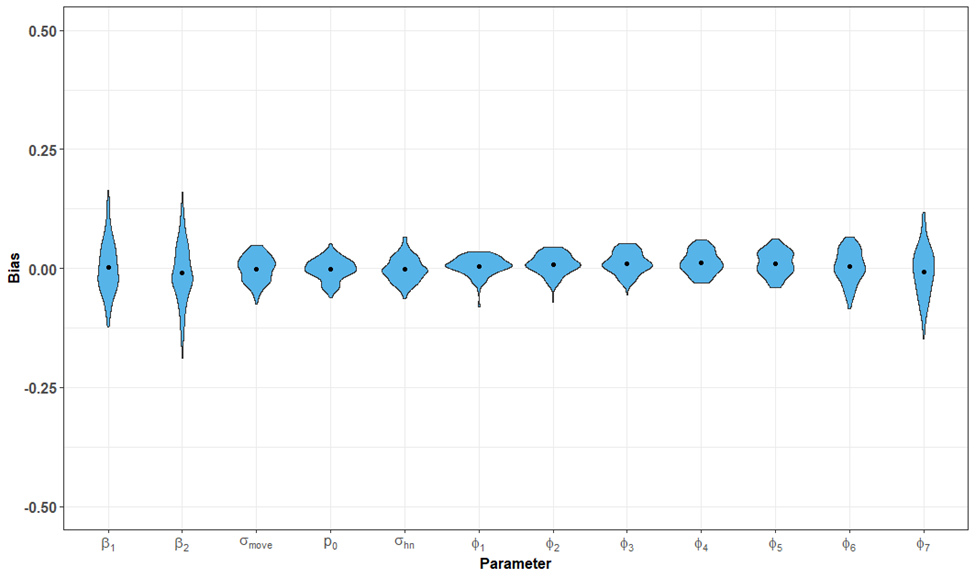


Figure S1. Violin plot showing the density distribution of bias for model parameters from 100 simulated datasets. Mean bias for each parameter shown as black circle.


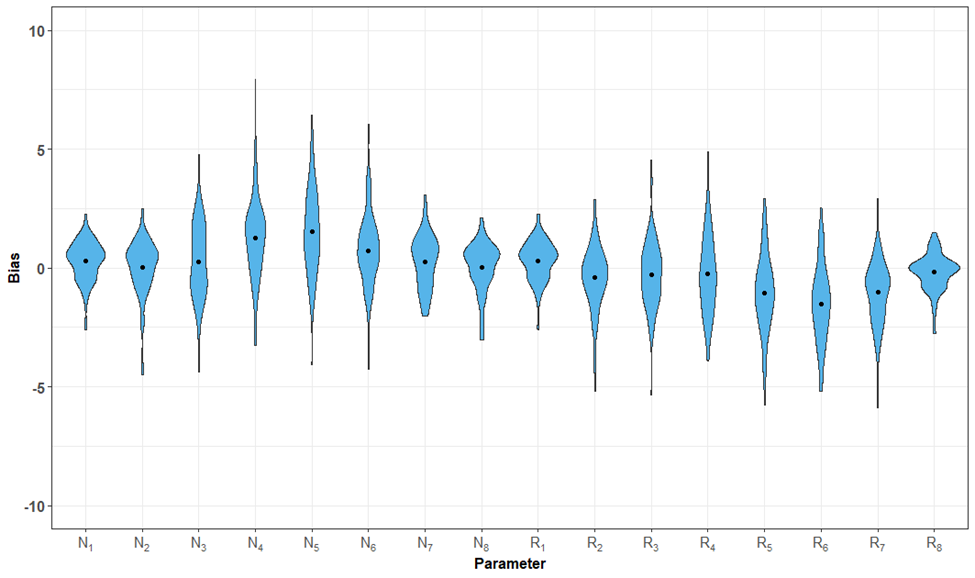


Figure S2. Violin plot showing the density distribution of bias for abundance, $N$, and number of recruits, $R$, in each primary period from 100 simulated datasets. Mean bias for each parameter shown as black circle. Note different y-axis scales in Figures S1 and S2.


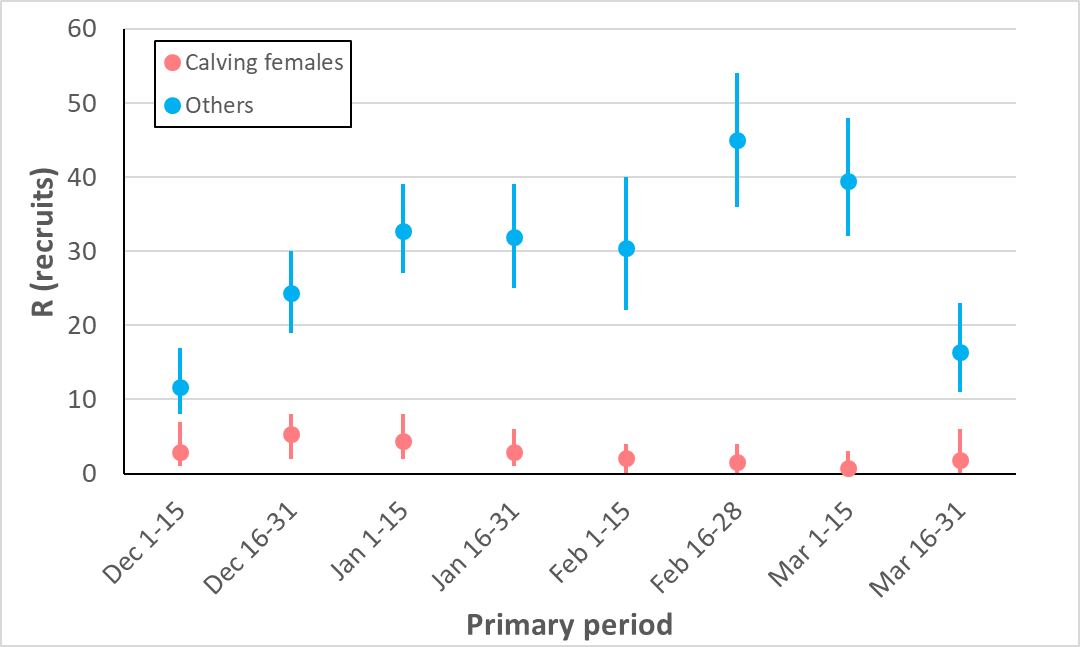


Figure S3. Estimated number (with 95% credible intervals) of calving female and all other right whales that entered the study area for each primary period.


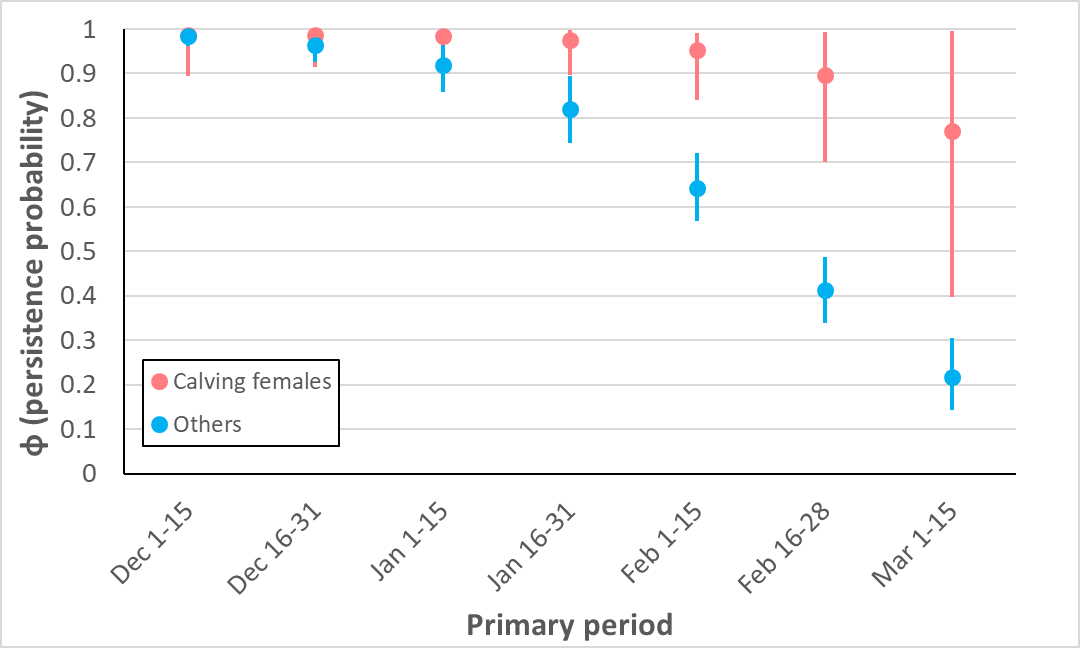


Figure S4. Estimated probability (with 95% credible intervals) of remaining in the study area by primary period for calving female and all other right whales.
